# *Lactobacillus gasseri* CBT LGA2 alleviates muscle protein degradation and inflammation in immobilization-induced mouse

**DOI:** 10.3389/fmicb.2025.1728172

**Published:** 2026-01-07

**Authors:** Ji Hun Jang, So Jeong Lim, Hyo Su Choi, Sanghyun Lim, Nam Su Oh

**Affiliations:** 1 Department of Food and Biotechnology, Korea University, Sejong, Republic of Korea; 2 Department of Food Regulatory Science, Korea University, Sejong, Republic of Korea; 3 R&D Center, Cell Biotech, Co., Ltd., Gimpo, Gyeonggi, Republic of Korea

**Keywords:** *Lactobacillus gasseri*, probiotics, muscle atrophy, inflammation, hindlimb immobilization, sarcopenia

## Abstract

**Introduction:**

Hindlimb immobilization rapidly induces skeletal muscle atrophy by reducing mechanical loading and accelerating proteolytic activity. This atrophy is further exacerbated by inflammatory signaling, which amplifies FOXO3a-driven expression of Atrogin-1 and MuRF1 and suppresses myogenic capacity. Emerging evidence suggests that specific probiotic strains may counteract these catabolic and inflammatory responses, prompting the evaluation of *Lactobacillus gasseri* CBT LGA2 (LGA2) in this study.

**Methods:**

In the present study, five probiotic strains were screened in C2C12 myotubes and RAW264.7 macrophages to assess anti-proteolytic and anti-inflammatory activities. Whole-genome sequencing was conducted to determine genetic safety and functional gene profiles. In vivo efficacy was evaluated using a hindlimb immobilization mouse model administered with LGA2 (1 × 10^⁸^ CFU/kg/day, 3 weeks), followed by assessments of muscle mass, grip strength, fiber morphology, and molecular markers.

**Results:**

LGA2 showed the strongest suppression of dexamethasone-induced muscle protein degradation and lipopolysaccharides-induced inflammatory responses among the screened strains. Genomic analysis identified genes related to antioxidant defense, immune modulation, and muscle protection. In immobilized mice, LGA2 significantly improved grip strength, preserved muscle mass, and restored muscle fiber cross-sectional area. Mechanistically, LGA2 maintained FOXO3a phosphorylation, reduced Atrogin-1 and MuRF1 expression, and recovered myogenin and MyHC isoforms (IIa, IIx, IIb). Additionally, LGA2 lowered TNF-α, IL-6, iNOS, and COX-2 levels while restoring IL-10 in muscle and serum.

**Discussion:**

These findings demonstrate that LGA2 mitigates disuse-induced muscle atrophy through coordinated anti-inflammatory, anti-proteolytic, and pro-myogenic mechanisms. Its genomic safety and multifunctional efficacy support LGA2 as a promising probiotic intervention for muscle health.

## 1 Introduction

Skeletal muscle atrophy, defined as the loss of muscle mass, occurs in aging, disuse, and chronic disease (Zhu et al., 2023). In older adults (typically aged 65 years and above), a progressive form of muscle wasting known as sarcopenia is recognized as a geriatric syndrome characterized by a decline in muscle mass accompanied by reduced muscle function (strength or performance) (Papadopoulou, 2020). The prevalence of sarcopenia rises with age; it is estimated to affect roughly 10% of community-dwelling older adults and up to 30–50% of more vulnerable populations such as nursing home residents or hospitalized seniors (Witham and Aihie Sayer, 2016). Beyond aging, muscle atrophy can also be induced by physical inactivity, such as prolonged bed rest or limb immobilization, which leads to significant reductions in muscle mass and fiber size even over short periods (Parker et al., 2022).

Muscle mass is maintained by a dynamic balance between protein synthesis and degradation, and tipping this balance toward degradation leads to atrophy (Wang et al., 2022). A principal pathway driving muscle protein breakdown is the ubiquitin–proteasome system (UPS). Under atrophic conditions, muscle-specific E3 ligases, Atrogin-1 and MuRF1, are upregulated, targeting structural proteins for proteasomal degradation (Haberecht-Muller et al., 2021; Zhang et al., 2020). These “atrogenes” are transcriptionally regulated by the forkhead box O (FOXO) family, particularly FOXO3a (Gellhaus et al., 2023).

When anabolic signaling is low, FOXO3a becomes activated and induces Atrogin-1 and MuRF1 expression, promoting rapid proteolysis. Consistently, increased FOXO3a and MuRF1 expression has been observed in muscle under conditions of disuse or nutrient deprivation (Sartori et al., 2021). Phosphorylation of FOXO3a by Akt inhibits its transcriptional activity, whereas dephosphorylation allows its nuclear translocation and activation, leading to increased expression of Atrogin-1 and MuRF1. In parallel with increased protein breakdown, anabolic and regenerative processes are suppressed during sarcopenia. Myogenesis is impaired due to downregulation of key regulatory factors such as MyoD and myogenin, which are essential for the differentiation of muscle precursor cells (Zammit, 2017). This leads to reduced formation of mature muscle proteins, including myofibrillar proteins like myosin heavy chain (MyHC). Consequently, muscle atrophy models often show declines in MyHC content suppressed expression of MyoD and myogenin, indicating an inability to replace or repair lost fibers (Sartori et al., 2021).

Inflammation further accelerates skeletal muscle catabolism. Pro-inflammatory cytokines, particularly tumor necrosis factor-α (TNF-α) and interleukin-6 (IL-6), exacerbate proteolysis by activating the NF-κB pathway and associated catabolic signaling cascades (Sartori et al., 2021). These cytokines can also impair the anabolic insulin/Akt axis, relieving inhibition on FOXO3a and thus amplifying Atrogin-1/MuRF1 expression (Ji et al., 2022). This inflammatory catabolic environment is especially prominent in disuse-induced muscle atrophy, as seen in hindlimb immobilization (HI) models, where reduced mechanical loading and increased cytokine levels synergistically promote FOXO3a activation, enhanced proteolysis, and muscle fiber shrinkage.

Recent research has increasingly highlighted the potential of probiotics to improve skeletal muscle health, particularly under atrophic conditions such as aging or disuse (Zhu et al., 2025). In particular, various *Lactobacillus* strains have demonstrated anti-atrophic effects. These probiotics can attenuate chronic inflammation and proteolytic signaling in muscle, thereby preserving muscle protein content and function (Ji et al., 2022). For instance, *Lactobacillus paracasei* PS23 supplementation in aged mice significantly decelerated muscle loss, accompanied by lower levels of inflammatory cytokines and oxidative stress markers (Chen et al., 2022). Certain *Lactobacillus* strains also directly modulate atrophy-related pathways: one recent study showed that feeding aged mice with *L. paracasei* markedly suppressed the activation of NF-κB and FOXO3a in muscle, reducing the expression of MuRF1 and Atrogin-1 while restoring myosin heavy chain (MyHC) and myogenin levels (Baek et al., 2023; Lee et al., 2021).

In this study, five probiotic strains were initially screened *in vitro* using DEX-treated C2C12 myotubes to assess anti-catabolic effects and LPS-stimulated RAW264.7 macrophages to evaluate anti-inflammatory activity. Based on these results, *Lactobacillus gasseri* CBT LGA2 (LGA2) demonstrated the most pronounced activity, effectively reducing proteolytic gene expression in C2C12 myotubes and inflammatory mediators in LPS-stimulated RAW264.7 cells. Based on these results, LGA2 was selected for whole-genome analysis and subsequent *in vivo* validation using a HI-induced muscle atrophy model. Muscle mass, strength and key markers of proteolysis, inflammation, and myogenesis were evaluated.

## 2 Materials and methods

### 2.1 Preparation of probiotics

Five lactic acid bacteria strains (*L. gasseri* IR13, LGA2, and *Lacticaseibacillus rhamnosus* IM14, IM18, and IM19), previously isolated from healthy infant feces, were used in this study (Jang et al., 2024). The isolates were identified by 16S rRNA gene sequencing (Macrogen Inc., Seoul, Korea), and the sequences were compared with reference sequences in the NCBI GenBank database for confirmation (>99% similarity). All strains were cultured twice in de Man, Rogosa, and Sharpe (MRS) broth (Difco Laboratory, MI, United States) at 37 °C for 21 h. After incubation, bacterial cells were harvested, washed three times with sterile saline (0.85% NaCl), and resuspended in sterile saline for subsequent experiments. After *in vitro* test, the strain *L. gasseri* LGA2 has been deposited in the Korean Federation of Culture Collections (KFCC, accession no. KFCC11983P).

### 2.2 Cell culture

C2C12 myoblasts and RAW264.7 macrophages were purchased from American Type Culture Collection (Manassas, VA, United States). Both cell lines were maintained in Dulbecco’s Modified Eagle’s Medium (DMEM; Gibco, Thermo Fisher Scientific, Waltham, MA, United States) supplemented with 10% fetal bovine serum (FBS; Gibco) and 1% penicillin–streptomycin (P/S; Gibco). Cells were cultured at 37 °C in a humidified atmosphere containing 5% CO₂.

### 2.3 C2C12 differentiation and atrophy model

C2C12 myoblasts were induced to differentiate upon reaching 90–100% confluence by switching to differentiation medium (DM) composed of DMEM supplemented with 2% horse serum (Gibco) and 1% P/S. After 4 days of differentiation, the resulting myotubes were treated with 10^6^ CFU/mL of each probiotic strain in antibiotic-free DM for 48 h. To induce muscle atrophy, dexamethasone (Sigma-Aldrich, St. Louis, MO, United States) was added to the medium at a final concentration of 100 μM for 24 h.

### 2.4 LPS-stimulated RAW 264.7 cells and nitric oxide assay

The production of nitric oxide (NO) by LPS-stimulated RAW 264.7 macrophages was evaluated using the Griess reaction. Briefly, RAW 264.7 cells were seeded at a density of 9 × 10^4^ cells per well in 96-well plates and allowed to adhere for 24 h. Cells were then preincubated with probiotic strains (10^5^ CFU/mL) for 2 h, followed by stimulation with lipopolysaccharide from *Escherichia coli* O111: B4 (Sigma-Aldrich, L2630) at a final concentration of 100 ng/mL for an additional 16 h. After treatment, the culture supernatants were collected and mixed with Griess reagent (equal volumes of 1% sulfanilamide and 0.1% N-(1-naphthyl) ethylenediamine dihydrochloride in 5% phosphoric acid) at a ratio of 1:2 (supernatant:reagent). The absorbance was measured at 540 nm using a microplate reader, and NO concentrations were determined from a sodium nitrite standard curve.

### 2.5 Western blot

C2C12 myotubes and gastrocnemius muscles were lysed in radioimmunoprecipitation assay (RIPA) buffer (Thermo Fisher Scientific) supplemented with phosphatase and protease inhibitor cocktails (PhosSTOP EASY pack and cOmplete™, Roche Diagnostics, Basel, Switzerland). The lysates were centrifuged at 18,000 × g for 20 min at 4 °C, and protein concentrations were determined using the Pierce™ BCA Protein Assay Kit (Thermo Fisher Scientific). Equal amounts of protein (30 μg) were separated by 10% SDS-PAGE and transferred onto polyvinylidene difluoride (PVDF) membranes (Millipore, Billerica, MA, United States). Membranes were blocked with 5% skim milk in TBST for 1 h at room temperature and then incubated overnight at 4 °C with primary antibodies diluted 1:1,000 in 5% bovine serum albumin (BSA). The following antibodies were used: phospho-FOXO3a (Ser253, #9465) and FOXO3a (#12829) (Cell Signaling Technology, Danvers, MA, United States); Atrogin-1 (sc-166806) and MuRF1 (sc-398608) (Santa Cruz Biotechnology, Dallas, TX, United States); and β-actin (sc-47778) (Santa Cruz Biotechnology); and GAPDH (GT239) (Genetex, Irvine, CA, United States). After washing with TBST, membranes were incubated with horseradish peroxidase (HRP)-conjugated secondary antibodies—anti-rabbit IgG (#7074S; Cell Signaling Technology) and anti-mouse IgG (GTX213111–01; GeneTex), diluted 1:2,000 in TBST for 1 h at room temperature. Protein bands were visualized with an enhanced chemiluminescence detection reagent (Bio-Rad Laboratories, Hercules, CA, United States) and imaged using a ChemiDoc MP system (Bio-Rad). Band intensities were quantified with ImageJ software (version 4.16; NIH, Bethesda, MD, United States) and normalized to GAPDH and β-actin as housekeeping proteins.

### 2.6 Reverse transcription–polymerase chain reaction (qRT-PCR)

The mRNA expression was measured by qRT-PCR as previously described (Jang et al., 2024). Briefly, total RNA was extracted using TRIzol reagent (Invitrogen, Carlsbad, CA, United States) and reverse-transcribed into cDNA using the BioFACT 2X Reverse Transcription Pre-Mix (BioFACT, Daejeon, Korea). Gene expression was analyzed using the 2^−^ΔΔCT method, and primer sequences are listed on Supplementary Table 1.

### 2.7 Whole genome analysis

The whole genome of LGA2 was *de novo* assembled using PacBio RS II (Pacific Biosciences, CA, United States) and Illumina HiSeq 2000 (Illumina, CA, United States) sequencing platforms. The sequencing analysis was conducted by Macrogen, Inc. (Seoul, Korea). Long-read data were assembled using the HGAP 3.0 pipeline within SMRT Analysis v2.3.0, and short reads from Illumina were mapped onto the assembled contigs for error correction using Pilon v1.21, resulting in a high-quality draft genome (Walker et al., 2014). Genomic features including coding DNA sequences (CDS), rRNA and tRNA genes, and GC content were annotated through sequence homology-based analysis. A circular genome map was generated based on contig annotations, and functional classification of genes was performed using the clusters of orthologous groups (COG) database (Tatusov et al., 2000). Screening for virulence-associated genes was performed using ABRicate with the Virulence Factor Database (VFDB) to identify potential virulence factors in the genome. Hits showing ≥ 80% nucleotide sequence identity and ≥ 70% alignment coverage were considered significant for the presence of virulence-related genes (Feldgarden et al., 2019).

### 2.8 Comparative genomics

The complete genome sequences of four *L. gasseri* reference strains, ATCC 33323 (GCF_000014425.1), 4 M13 (GCF_002158885.1), HL70 (GCF_017840575.1), and EJL (GCF_013363915.1), were retrieved from the NCBI database based on their high sequence similarity to LGA2. These genomes were subjected to comparative genomic analysis. Pan-genome profiling was performed using Roary v1.007001 to identify core and accessory genes, and core gene alignments were used to construct a phylogenetic tree with FastTreeMP v2.1.11, revealing the genomic relatedness among the strains. Additionally, gene content was visualized through a presence/absence dendrogram and a Venn diagram based on pan-genome orthologous groups (POGs).

### 2.9 Animal and experimental design

**Figure 1:**
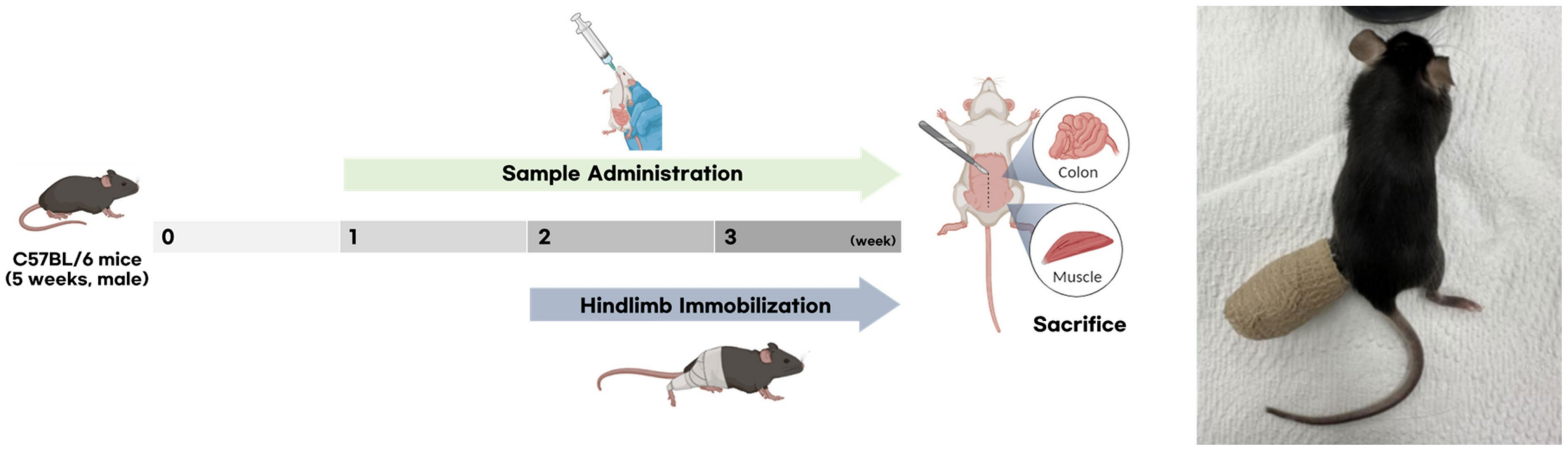
The experimental design of the preclinical study.

Based on the results of *in vitro* screening, LGA2 was selected for *in vivo* experiments. The LGA2 used in this study was manufactured by Cell Biotech (Gimpo, Gyeonggi, Korea). Five-week-old male C57BL/6 J mice were obtained from Samtako Biokorea (Osan, Korea) and housed under controlled conditions (25 ± 2 °C, 60% relative humidity, 12 h light–dark cycle) with *ad libitum* access to food and water. As hindlimb-immobilized mice could not reliably access the food rack, a fixed amount of standard chow was provided directly on the cage floor. Based on the typical intake of adult C57BL/6J mice (approximately 4 g per mouse per day), 16 g of chow was supplied per cage per day to ensure equal accessibility across groups. The study protocol was approved by the Institutional Animal Care and Use Committee (IACUC) of Korea University (Approval No. KUIACUC-2025-0029). After a one-week acclimation period, the mice were randomly assigned to three groups (*n* = 8 per group). LGA2 (1 × 10^8^ CFU/kg/day) was orally administered daily in an equal volume of vehicles. The experiment lasted for 3 weeks, during which LGA2 was administered daily throughout the study period. The experimental protocol and hindlimb immobilization (HI) procedure were conducted according to previously described methods (You et al., 2015), with minor modifications as referenced in the cited literature. At the end of the experiment, mice were anesthetized, and gastrocnemius and quadriceps muscles were collected, weighed, and either snap-frozen in liquid nitrogen for molecular analyses or fixed in 10% neutral-buffered formalin for histological examination. The overall experimental design is illustrated in Figure 1.

### 2.10 Measurement of grip strength

Grip strength was measured weekly throughout the experimental period using a grip strength meter (Bioseb, Chaville, France). Mice were gently lifted by the tail and allowed to grasp the grid with both forelimbs and hindlimbs. Once a firm grip was established, each mouse was pulled horizontally until it released the grid, and the peak force was automatically recorded. Each mouse performed five consecutive trials, and the mean value was used for analysis. Grip strength values were normalized to body weight. All measurements were conducted by an investigator blinded to the group assignments to ensure unbiased assessment.

### 2.11 Histological analysis

Gastrocnemius muscles were fixed in 4% paraformaldehyde, embedded in paraffin, and sectioned at 4 μm thickness. Sections were stained with hematoxylin and eosin (H&E) for 13 h and visualized under a light microscope (Olympus, Tokyo, Japan). Cross-sectional areas (CSA) of myofibers were measured using ImageJ software (NIH, Bethesda, MD, United States).

### 2.12 Enzyme-linked immunosorbent assay (ELISA)

Blood samples were collected via cardiac puncture under anesthesia and allowed to coagulate at room temperature for 5–20 min. The samples were then centrifuged at 1300 × g for 15 min, and the supernatant serum was collected for cytokine analysis. IL-6 and TNF-*α* levels were measured by ELISA MAX Deluxe Sets (BioLegend, San Diego, California, United States) according to the manufacturer’s instructions. Absorbance was measured at 540 nm using a microplate reader.

### 2.13 Statistical analysis

All results are presented as the mean ± standard deviation (SD). Statistical analyses were performed using SPSS software version 25.0 (IBM, Chicago, IL, United States). Group differences were assessed using one-way analysis of variance (ANOVA), followed by Duncan’s multiple comparison test. Statistical significance was set at *p* < 0.05, unless otherwise stated.

## 3 Results

### 3.1 Pretreatment with *L. gasseri* LGA2 prevents ubiquitin-mediated muscle protein degradation in muscle cell

**Figure 2:**
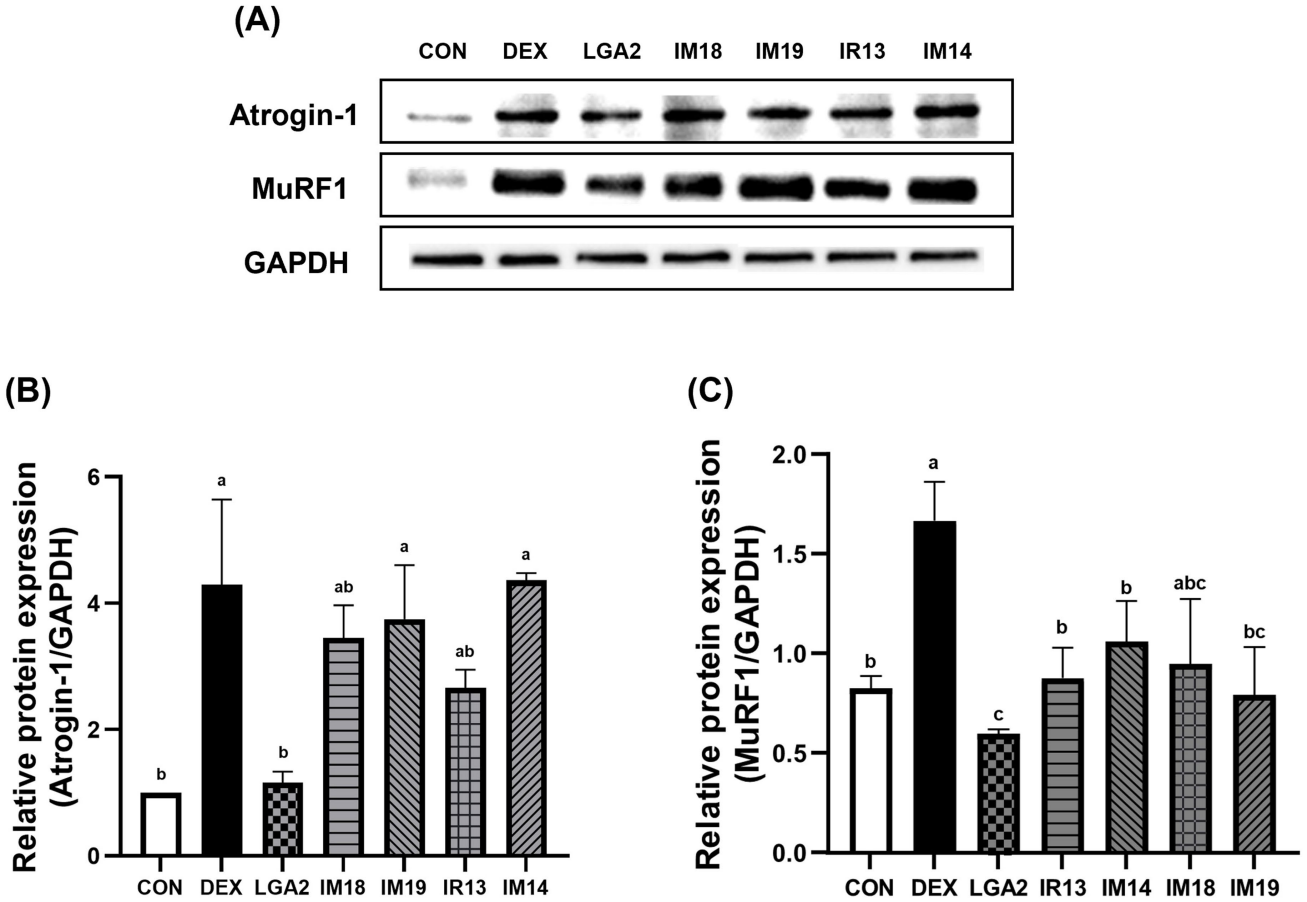
Effect of probiotics candidates on muscle protein degradation through ubiquitin proteasome pathway in dexamethasone-induced muscle atrophy. **(A)** Western blot analysis of Atrogin-1 and MuRF1. **(B,C)** Relative protein expression of Atrogin-1 and MuRF1. Protein levels were normalized to GAPDH as the housekeeping protein. Data represent mean ± SD of three independent biological replicates. Different letters (a–c) denote significant differences at *p* < 0.05.

To induce muscle atrophy, differentiated C2C12 myotubes were treated with 100 μM DEX. To assess the preventive effects of the probiotic candidates, five strains were pretreated to the myotubes prior to DEX exposure. The protein levels of Atrogin-1 and MuRF1, two key markers of ubiquitin-mediated protein degradation in skeletal muscle, were subsequently analyzed by Western blot (Figure 2A). The relative protein levels of Atrogin-1 and MuRF1 were significantly increased by DEX treatment (*p* < 0.05). However, pretreatment of LGA2 significantly reduced the expression of Atrogin-1 to levels comparable to the control group (*p* < 0.05, Figure 2B). In the case of MuRF1, pretreatment with LGA2, IR13, and IM14 significantly reduced its expression, with LGA2 showing the most pronounced effect among the strains (*p* < 0.05, Figure 2C). These results suggest that LGA2 can alleviate ubiquitin-mediated muscle protein degradation.

### 3.2 LGA2 attenuates nitric oxide production and LPS-induced inflammation in macrophages

**Figure 3:**
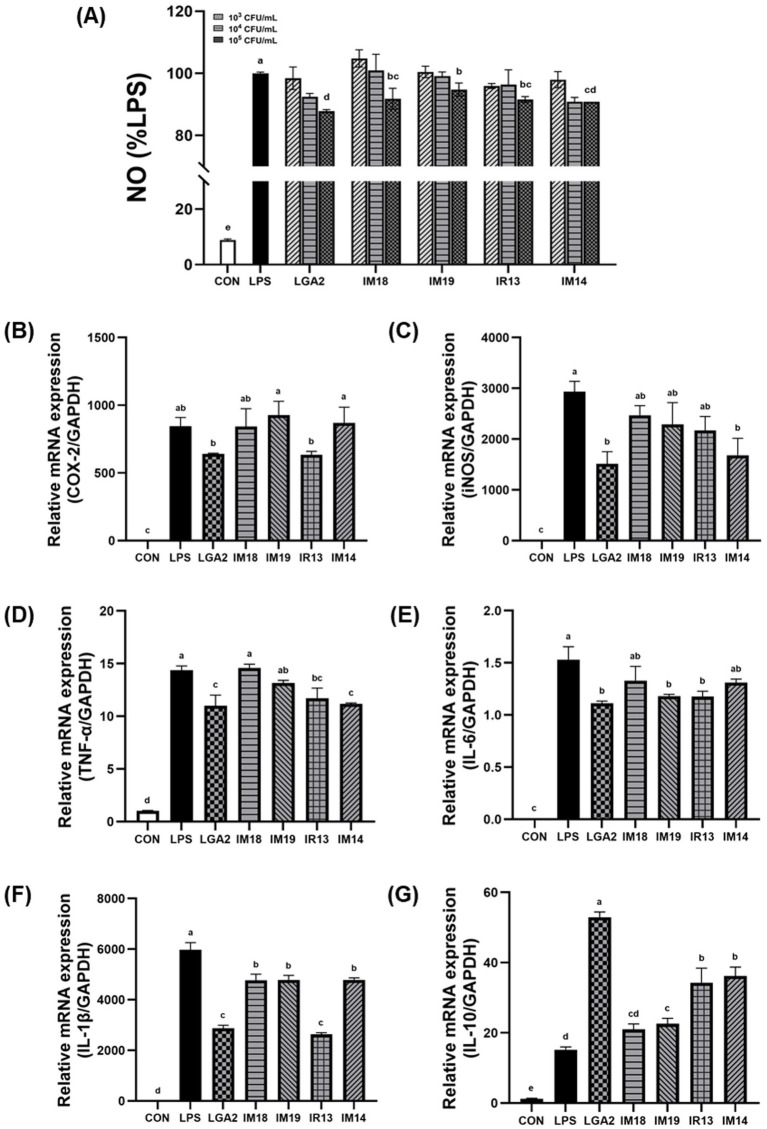
Anti-inflammatory effect of probiotic candidates LPS-induced in RAW264.7 macrophage cells. **(A)** NO production was evaluated using the Griess reagent. The mRNA expressions of inflammatory genes were measured by qRT-PCR and normalized using GAPDH as the housekeeping gene. **(B)** Relative mRNA expression COX-2 **(C)** iNOS **(D)** TNF-α **(E)** IL-6 **(F)** IL-1β, and **(G)** IL-10. Data represent mean ± SD of three independent biological replicates. Different letters (a–e) denote significant differences at *p* < 0.05.

To assess the effect of probiotic candidates on NO production, RAW264.7 macrophage cells were pretreated for 12 h with various concentrations (10^5^, 10^6^ and 10^7^ CFU/mL), followed by stimulation with LPS (100 ng/mL) for an additional 12 h (Figure 3). LPS treatment significantly increased NO production, however, pretreatment with probiotic candidates at 10^7^ CFU/mL significantly attenuated this response (*p* < 0.05, Figure 3A). Among the five strains, LGA2 treatment showed the most pronounced reduction in NO production (87.7%, *p* < 0.05). Furthermore, the mRNA expression of inflammatory cytokines such as COX-2, iNOS, TNF-α, IL-6, IL-1β and IL-10 in LPS-treated macrophages was assessed by qRT-PCR (Figures 3B–G). All the inflammatory cytokines were significantly increased by LPS stimulation (p < 0.05). While several probiotic candidates showed limited or selective effects on cytokine expression, LGA2 exhibited the most consistent anti-inflammatory activity, significantly reducing iNOS and TNF-α levels (*p* < 0.05). IR13 also reduced TNF-α and IL-6 levels, while IM14 had significant effects on iNOS and TNF-α, and IM19 on IL-6 (*p* < 0.05). Moreover, all strains except IM18 significantly increased the anti-inflammatory cytokine IL-10 (*p* < 0.05). Notably, LGA2 exhibited the most consistent and statistically significant anti-inflammatory effects among the five strains (*p* < 0.05). As LGA2 exhibited the strongest preventive effects on muscle degradation and inflammation *in vitro*, it was selected for further investigation using a HI-induced mouse model.

### 3.3 Genome features of LGA2

**Figure 4:**
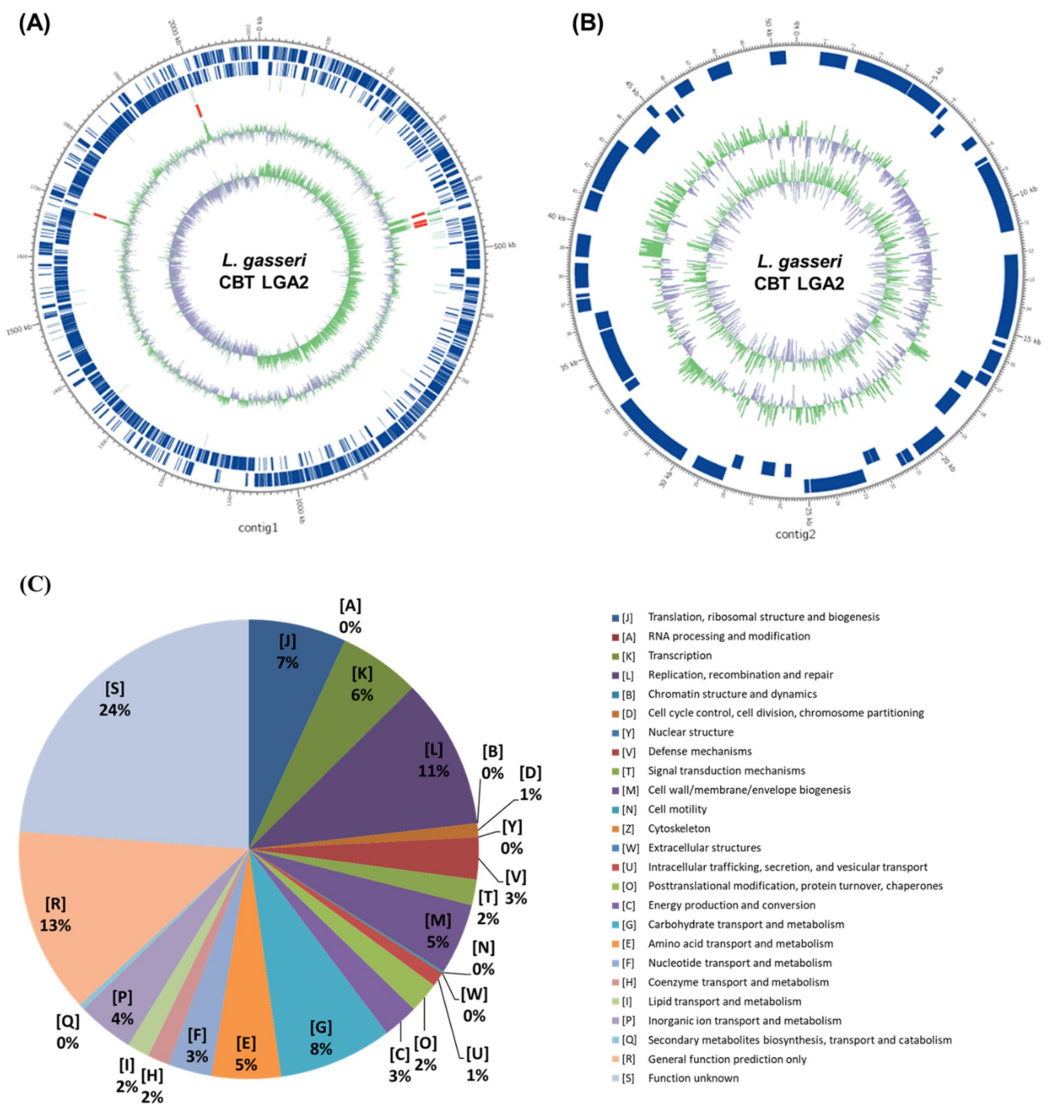
Circular maps of **(A)** contig 1 and **(B)** contig 2. The circular maps were drawn by applying the annotation results. Marked properties are shown from outside to the inside: CDS on forward, CDS on the reverse strand (the non-CDS region was described as blank), rRNA (red), tRNA (light green), GC content (light green peak outside, the area with higher GC% than average; interior lavender peak, other regions; the height of the peak represents the difference from the average GC%), and GC skew (according to the formula, (G-C)/(G + C), the light green peak outside represents the area with higher G content, while the lavender peak inside means the region with higher C content). **(C)** Functional categorization of all predicted ORFs in LGA2 genome.

The selected strain LGA2 was subjected to whole genome sequencing, resulting in a total of 729,332,865 bp from a 20-kb genomic library (Figure 4). Genome characteristics were presented in Table 1. The whole genome of LGA2 consists of total length of 3,774,653 bp, with a G + C content of 35.07%. The assembly resulted in two contigs, with the largest contig measuring 2,114,703 bp (Figure 4A) and the smallest 50,877 bp (Figure 4B). The N50 value was 2,114,703 bp, indicating a relatively high level of assembly continuity. A total of 2,251 genes were annotated, including 2,157 predicted CDSs. The genome also contains 78 tRNA genes and 15 rRNA genes. Circular genome maps were constructed based on contig annotation results (Figure 4C). Out of 2,157 predicted genes, 1,555 (77.3%) were assigned to COG, representing 18 functional categories. The most enriched categories included replication, recombination and repair (L; 216 ORFs), carbohydrate transport and metabolism (G; 163 ORFs), translation and ribosomal structure (J; 140 ORFs), transcription (K; 117 ORFs), and cell wall/membrane/envelope biogenesis (M; 102 ORFs). Notably, categories R (general function prediction only) and S (function unknown) were also highly represented. Furthermore, no virulence factors were detected in LGA2 through whole-genome sequencing analysis using ABRicate with the VFDB database.

**Table 1:** Genome characteristics.

Genome characteristics	
Assembly results	
Genome size (bp)	3,774,653
G + C content (%)	35.07
Number of contigs	2
N50 length (bp)	2,114,703
Shortest length (bp)	50,877
Largest length (bp)	2,114,703
Annotation results	
Annotated genes	2,251
Predicted CDS	2,157
Number of rRNA genes	15
Number of tRNA genes	78

### 3.4 Comparative genomics of LGA2 to other *Lactobacillus gasseri* strains

**Figure 5:**
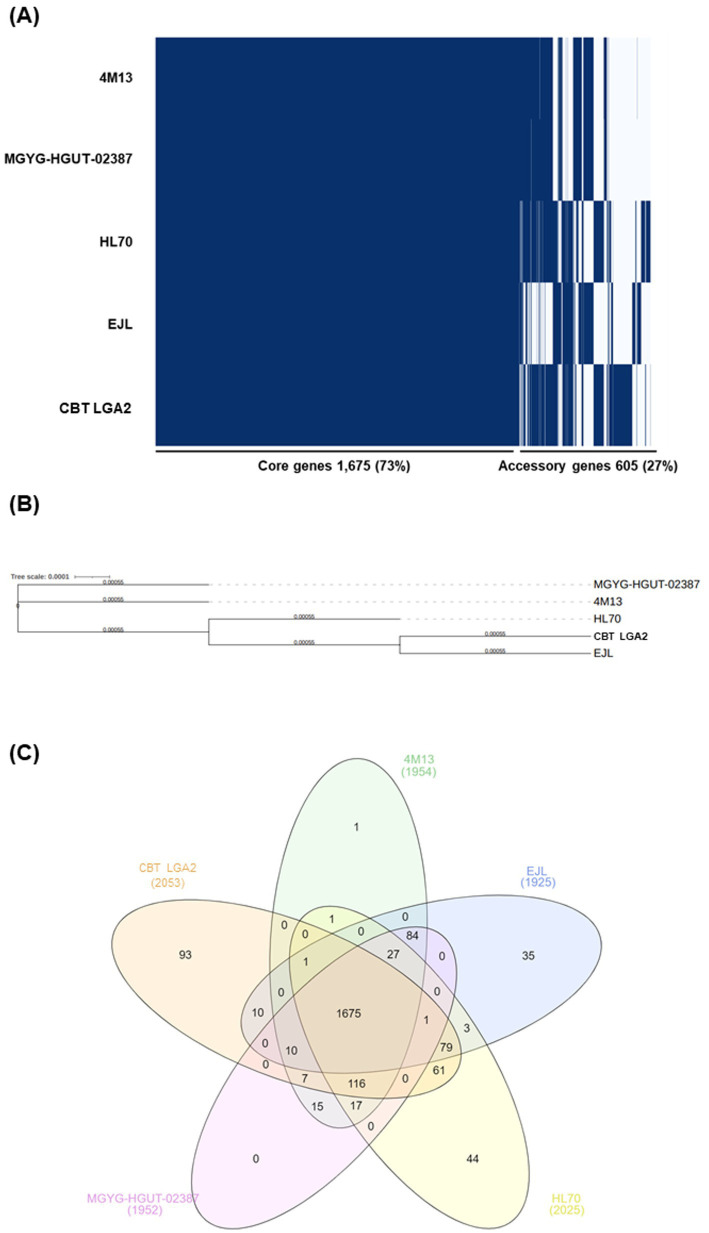
Comparative genomics of LGA2 compared with four reference strains: **(A)** Dendrogram based on presence/absence of POGs; **(B)** phylogenetic tree; **(C)** Venn diagram of five strains; the numbers marked in the figure show the number of POGs found among the genomes.

Comparative pan-genome analysis was conducted between LGA2 and four reference strains (Figure 5). A total of 2,745 gene clusters were identified, comprising 1,130 core genes and 1,615 shell genes, with no soft core or cloud genes detected. A presence/absence matrix of gene clusters was visualized (Figure 5A), and a phylogenetic tree was constructed based on core gene alignments generated by Roary using FastTreeMP v2.1.11 (Figure 5B). LGA2 exhibited a modest genetic divergence from the reference strains, with *L. gasseri* EJL clustering most closely with LGA2. As shown in Figure 5C, LGA2 shared 1,130 POGs with the reference strains and possessed 75 unique genes. These findings highlight the genomic novelty of the LGA2 strain.

### 3.5 LGA2 restores muscle mass and performance in HI-induced mice

**Figure 6:**
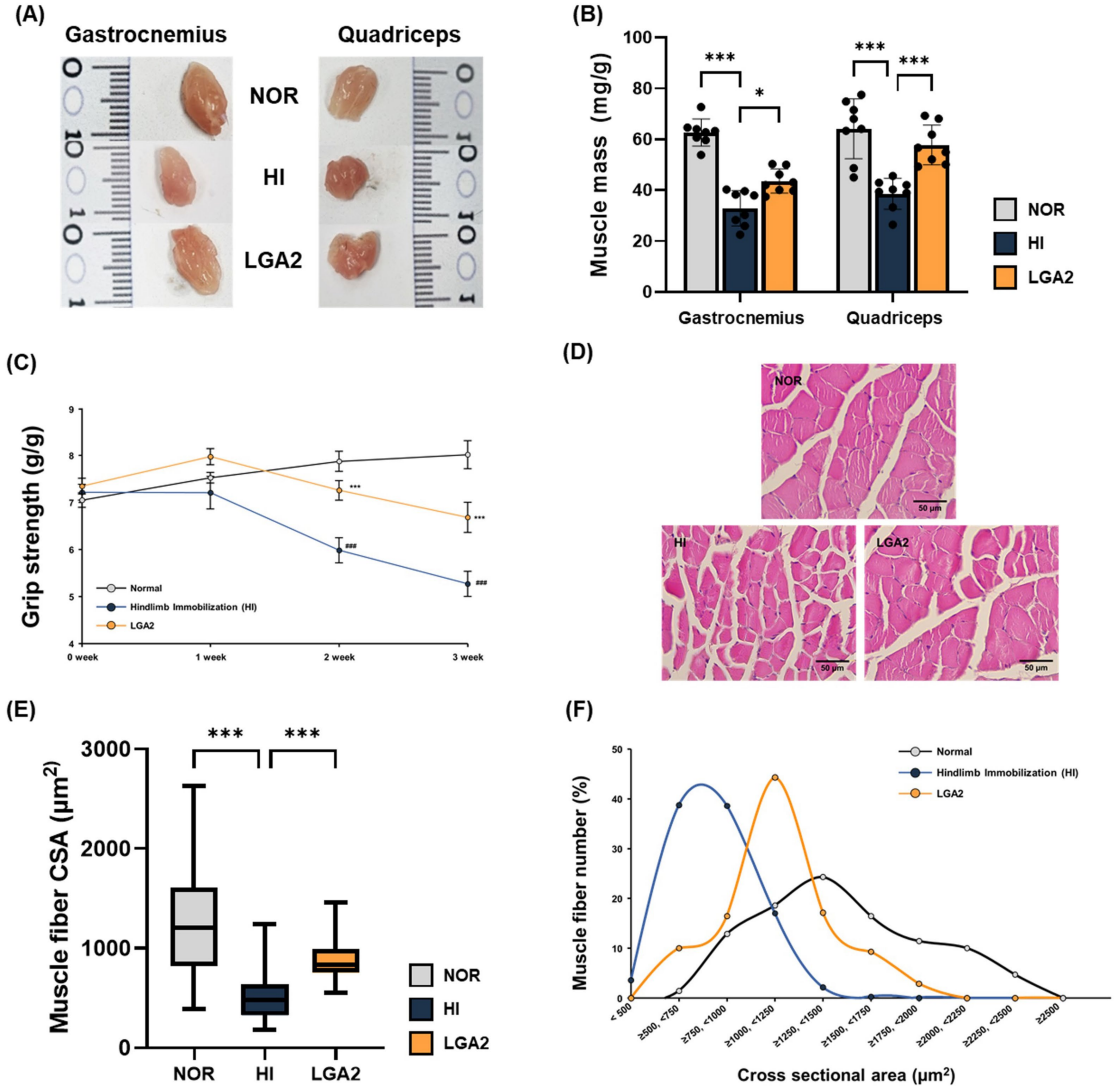
Effect of LGA2 on muscle and strength in hindlimb immobilization-induced mouse. **(A)** Representative photograph of gastrocnemius and quadriceps muscles from each group. **(B)** The weight of gastrocnemius and quadriceps muscle tissue. Muscle mass was normalized to body weight (g/g). **(C)** Grip strength curve. Grip strength was expressed as ratio to body weight (g/g). **(D)** Representative images of gastrocnemius of each group. Scale bars = 50 μm. **(E)** Comparison of muscle fiber cross sectional area (CSA). **(F)** The distribution graph of muscle fiber CSA (%). NOR, normal group; HI, hindlimb-immobilized group; LGA2, HI group treated with *Lactobacillus gasseri* CBT LGA2. Data represent mean ± SD of 8 biological replicates (individual mice) per group. *p < 0.05 and ***p < 0.001 represent significant differences between marked groups.

To evaluate the protective effect of LGA2 on disuse-induced muscle atrophy, gastrocnemius and quadriceps muscles were collected after 2 weeks of HI (Figure 6A). HI resulted in significant reduction in muscle mass, with gastrocnemius and quadriceps weights decreased by 47.5 and 39.8%, respectively, compared to the normal group (*p* < 0.001, Figure 6B). However, LGA2 treatment significantly increased gastrocnemius and quadriceps muscle mass by 32.5 and 49.7%, respectively, compared to the HI group (*p* < 0.001). Muscle strength, assessed weekly, was also notably preserved by LGA2 administration (Figure 6C). After 2 weeks of immobilization, grip strength declined by 24.0% in the HI group relative to the normal group, whereas the LGA2 group showed only an 8.0% reduction, indicating a significant preventive effect (*p* < 0.001). This difference persisted through week 3, with the HI group showing a 33.0% reduction in grip strength, while the LGA2 group showed only a 15.1% decrease compared to the normal group. In addition, the histological analysis was conducted by H&E staining and measuring muscle fiber CSA of each group (Figure 6D). CSA analysis revealed a 59.5% reduction in the HI group relative to the normal group, which was attenuated by 77.0% recovery in the LGA2-treated group (Figures 6E,F). Collectively, these results indicate that LGA2 administration effectively prevents muscle mass loss, strength decline, and fiber atrophy in a HI-induced sarcopenia model.

### 3.6 LGA2 suppresses FOXO3a-mediated ubiquitin–proteasome muscle protein degradation in HI-induced muscle atrophy

**Figure 7:**
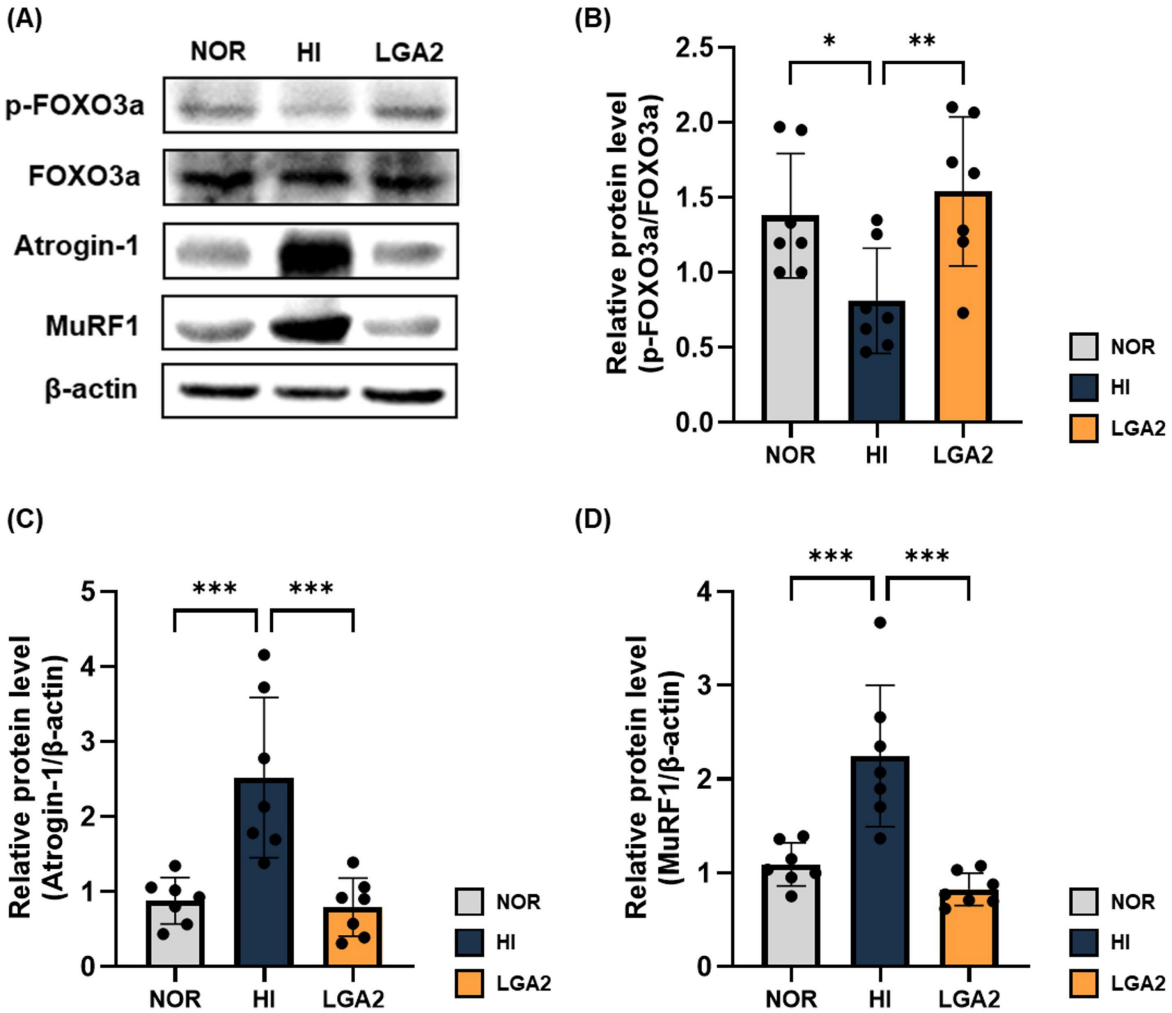
Preventive effect of LGA2 on muscle protein degradation in hindlimb immobilization-induced mouse. **(A)** The relative levels of proteins related to muscle protein degradation was determined by western blot and normalized to β-actin. **(B)** The phosphorylation ratio of FOXO3a. **(C)** Atrogin-1 **(D)** MuRF1. NOR, normal group; HI, hindlimb-immobilized group; LGA2, HI group treated with *Lactobacillus gasseri* CBT LGA2. Data represent mean ± SD of 7 biological replicates (individual mice) per group. *p < 0.05, **p < 0.01 and ***p < 0.001 represent significant differences between marked groups.

The regulation of muscle protein degradation through the FOXO3a signaling pathway was examined using Western blotting (Figure 7A). The phosphorylation level of FOXO3a was significantly reduced in the HI group (*p* < 0.05; Figure 7B), indicating dephosphorylation-mediated nuclear translocation and activation of its target E3 ubiquitin ligases, Atrogin-1 and MuRF1. Accordingly, the protein levels of Atrogin-1 and MuRF1 were markedly elevated in the HI group (*p* < 0.001; Figures 7C,D). In contrast, LGA2 treatment markedly restored FOXO3a phosphorylation (*p* < 0.01), thereby retaining FOXO3a in the cytoplasm and suppressing its transcriptional activity toward Atrogin-1 and MuRF1. Consequently, LGA2 significantly downregulated the expression of both E3 ligases (*p* < 0.001), returning their levels close to baseline. Collectively, these results indicate that LGA2 counteracts HI-induced activation of ubiquitin–proteasome-mediated muscle protein degradation by promoting cytoplasmic retention of phosphorylated FOXO3a and inhibiting its transcriptional activity.

### 3.7 LGA2 ameliorated myogenesis and inflammation in HI-induced mouse muscle

**Figure 8:**
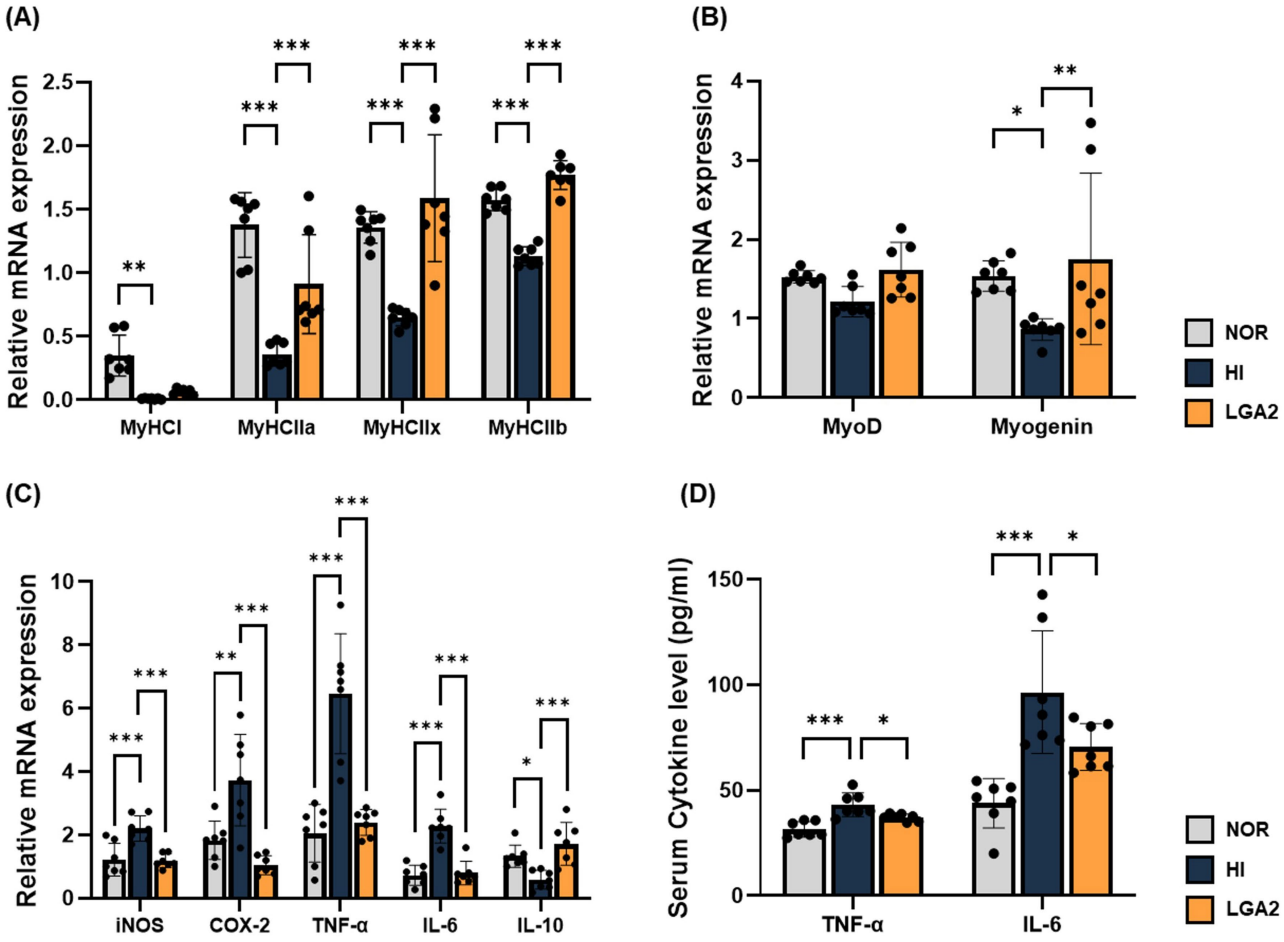
Preventive effect of LGA2 on myogenesis and inflammation in muscle of immobilization-induced mouse model. The gene expressions were measured using qRT-PCR and normalized to GAPDH. **(A)** MyHC series (I, IIa, IIx, IIb); **(B)** MyoD and myogenin; **(C)** iNOS, COX-2, TNF-α, IL-6, and IL-10. The levels of inflammatory cytokines were assessed by ELISA in serum. **(D)** Serum levels of TNF-α and IL-6. NOR, normal group; HI, hindlimb-immobilized group; LGA2, HI group treated with *Lactobacillus gasseri* CBT LGA2. Data represent mean ± SD of 7 biological replicates (individual mice) per group.

To determine whether LGA2 modulates myogenesis in hindlimb HI-induced muscle atrophy, the expression of key myogenic transcription factors was analyzed by qRT-PCR (Figures 8A,B). The expression of MyHC isoforms was markedly reduced in the HI group (*p* < 0.01), whereas LGA2 treatment significantly restored the expression of MyHC IIa, IIx, and IIb (*p* < 0.001; Figure 8A). Although MyoD expression remained unchanged, myogenin was significantly downregulated by HI (*p* < 0.05) and significantly recovered following LGA2 administration (*p* < 0.01; Figure 8B), indicating that LGA2 mitigates the suppression of myogenic transcription induced by disuse. To assess the anti-inflammatory potential of LGA2, inflammatory markers were measured in both muscle tissue and serum. In gastrocnemius muscle, HI significantly elevated the mRNA expression of iNOS, COX-2, TNF-*α*, and IL-6 (*p* < 0.01), which were all markedly suppressed by LGA2 (*p* < 0.01; Figure 8C). Conversely, IL-10 expression was significantly reduced in HI (*p* < 0.05) and restored to baseline or higher by LGA2 (*p* < 0.001). In the serum, TNF-α and IL-6 levels increased by 36.9 and 119.7% in the HI group, respectively (*p* < 0.001), whereas LGA2 significantly lowered these elevations by 14.1 and 26.9% (*p* < 0.05; Figure 8D). Collectively, these findings demonstrate that LGA2 confers both local and systemic anti-inflammatory effects and preserves myogenic potential under disuse-induced atrophy.

## 4 Discussion

Probiotics are increasingly being recognized for their beneficial effects beyond gut health, with mounting evidence supporting their role in skeletal muscle maintenance and function (Liu et al., 2021). In particular, *Lactobacillus* strains have been suggested to influence muscle function through several mechanisms, including the suppression of inflammatory responses, reduction of excessive reactive oxygen species (ROS), and modulation of skeletal muscle metabolism, all of which are central to the preservation of muscle mass and function (Zhu et al., 2025). In this study, five probiotic strains were assessed for their functional effects on muscle health using DEX-induced C2C12 myotubes and LPS-stimulated RAW264.7 macrophage models through *in vitro* screening, LGA2 was found to exhibit the most prominent effects, significantly suppressing DEX-induced muscle protein degradation and LPS-stimulated inflammatory responses. Based on these outcomes, LGA2 was selected as the most promising candidate for further genomic characterization and subsequent *in vivo* validation in a HI-induced muscle atrophy model.

Atrogin-1 and MuRF1 are critical muscle-specific E3 ubiquitin ligases that mediate UPS-dependent protein degradation, playing a central role in skeletal muscle atrophy (Zhang et al., 2020). Their expression is primarily regulated by the FOXO transcription factors, which are activated under catabolic conditions, leading to accelerated muscle protein breakdown. Treatment with DEX, a synthetic glucocorticoid, significantly induces the transcription of Atrogin-1 and MuRF1 in C2C12 myotubes, thereby promoting proteolytic activity and inducing a cellular environment characteristic of muscle atrophy (Jang et al., 2024). Recent study demonstrated that conditioned medium from *L. rhamnosus* JY02 attenuated DEX-induced myotube atrophy by downregulating MuRF1 and Atrogin-1 expression in C2C12 cells, highlighting the therapeutic potential of probiotic-derived factors in muscle preservation (Lee et al., 2021). Similarly, LGA2 pretreatment significantly inhibited the DEX-induced upregulation of these genes, indicating its ability to preserve muscle protein homeostasis by modulating catabolic signaling.

To investigate the anti-inflammatory properties of LGA2 in a context relevant to muscle catabolism, its effects were assessed in LPS-stimulated RAW264.7 macrophages. LPS stimulation markedly elevated nitric oxide (NO) production and upregulated the expression of inflammatory mediators (COX-2 and iNOS) and pro-inflammatory cytokines (TNF-α, IL-6, IL-1β), while concurrently suppressing the anti-inflammatory cytokine IL-10 (Wang et al., 2023). In previous study, several *Lactobacillus* strains have been shown to attenuate LPS-induced inflammation in RAW264.7 cells by suppressing NO production and downregulating COX-2, TNF-α, IL-1β, and IL-6 expression (Khanna et al., 2020). Consistent with these findings, LGA2 demonstrated comparable anti-inflammatory activity, further supporting its potential as a probiotic strain with immunomodulatory capacity. These dual effects on muscle catabolism and inflammatory signaling formed the basis for selecting LGA2 through *in vitro* screening as the most promising candidate for subsequent genomic and *in vivo* validation.

To further elucidate the genetic basis underlying these functional effects, whole-genome sequencing of LGA2 was carried out, followed by comparative analysis with publicly available *Lactobacillus* genomes. Based on 16S rRNA phylogenetic relationships, average nucleotide identity (ANI), and the presence or absence of POGs, LGA2 was identified as a novel genomic variant within the *Lactobacillus* genus. Genomic analysis of LGA2 identified key genes associated with peptide transport and oxidative stress defense, offering mechanistic insights into its physiological effects. Notably, the genome encoded dtpT (Gene 1,682), a proton motive force-driven di−/tripeptide transporter, as well as oppD (Gene 1,543) and oppF (Gene 1,542), components of the ATP-binding cassette (ABC) oligopeptide permease system. These transporters facilitate the uptake of dietary peptides, including bioactive peptides with functional roles in host physiology (Oh et al., 2018). In addition, Genes involved in oxidative stress defense were also present, including trxA (Gene 1794) and trxB (Gene 727), which constitute the core of the thioredoxin system responsible for the reduction of reactive oxygen and nitrogen species via thiol-dependent peroxidase reactions (Lu and Holmgren, 2014). Additionally, npr (Gene 1,058), encoding NADH peroxidase, was detected as a contributor to NADH-dependent ROS detoxification mechanisms. These genetic features suggest that LGA2 may exert its protective effects through enhanced uptake of functional peptides and reinforcement of antioxidative defense systems, ultimately contributing to muscle preservation and anti-inflammatory responses (Kunji et al., 1995; Naraki et al., 2020; Yang et al., 2024). Notably, screening of the whole-genome sequencing data using ABRicate with the VFDB database did not identify any virulence factors, indicating the absence of known pathogenicity-related genes in LGA2 (Feldgarden et al., 2019). The genomic information obtained from LGA2 has provided valuable insight into the molecular basis of its probiotic properties and offers a foundation for its potential application in functional food.

In addition to these genomic characteristics, the functional genes identified in LGA2 offer further context for understanding potential mechanisms underlying its anti-catabolic and anti-inflammatory actions. The presence of peptide transport–associated genes (dtpT, oppD, and oppF) indicates that LGA2 may participate in the uptake or handling of bioactive peptides, a process that has been implicated in the functional activity of several *Lactobacillus* strains (Fasano et al., 2019). Similarly, antioxidative genes, including trxA, trxB, and npr, are closely linked to cellular redox regulation, which is known to influence FOXO3a signaling and ubiquitin–proteasome–mediated protein degradation (Lu and Holmgren, 2014; Tagliazucchi et al., 2019). Although these mechanistic pathways were not directly examined in the present study, the genomic features of LGA2 provide a biologically relevant framework for interpreting the observed *in vivo* responses and delineate avenues for future mechanistic investigations.

Sarcopenia can result from various conditions such as prolonged muscle inactivity, neural impairment, chronic disease, and aging, with reduced physical activity recognized as a major contributing factor (Fanzani et al., 2012; Cohen et al., 2015). Among several established *in vivo* models for sarcopenia, including aging and hindlimb unloading, the HI model was adopted in this study to simulate disuse-induced muscle atrophy under controlled conditions (You et al., 2015). This model was particularly suitable for evaluating the efficacy of LGA2, as prolonged immobilization is known to trigger skeletal muscle atrophy through the activation of proteolytic pathways such as the ubiquitin-proteasome system, leading to disruption of muscle protein homeostasis. Inflammation has also been suggested to contribute directly to the loss of muscle mass and function associated with sarcopenia, as immobilization induces local inflammatory responses and increases the expression of pro-inflammatory cytokines in the affected muscle tissue (Caron et al., 2009; Buchmann et al., 2022). Given the previously observed anti-catabolic, anti-inflammatory, and antioxidant effects of LGA2 *in vitro*, the HI model was considered an appropriate system to validate its efficacy in preserving muscle integrity under disuse conditions.

Previous studies have demonstrated that HI leads to significant declines in muscle mass, grip strength, and muscle fiber CSA (Shin et al., 2020; Rahman et al., 2025; Chacon-Cabrera et al., 2016). Consistent with these findings, the present study confirmed that 2 weeks of immobilization markedly reduced muscle mass in the gastrocnemius and quadriceps, decreased grip strength, and induced substantial fiber atrophy. These reductions in muscle mass and CSA are likely attributable to inflammation-induced enhancement of proteolytic activity, which disrupts muscle protein homeostasis and accelerates muscle wasting (Costamagna et al., 2015; Gao et al., 2018). Notably, oral administration of LGA2 prior to immobilization resulted in a modest improvement in grip strength during the first week of treatment. Following immobilization, LGA2-treated mice exhibited a significantly attenuated decline in muscle strength. In addition, gastrocnemius and quadriceps muscle mass were markedly increased, and CSA partially recovered compared to the untreated group.

Skeletal muscle mass is tightly regulated by the balance between anabolic and catabolic pathways, with the UPS playing a central role in muscle protein degradation (Zhang et al., 2020; Jeon and Choung, 2021). Under disuse conditions such as immobilization, FOXO3a is dephosphorylated and translocated into the nucleus, where it activates the transcription of E3 ubiquitin ligases, including Atrogin-1 and MuRF1, thereby promoting proteolysis (Sartori et al., 2021; Huang et al., 2022). Increased expression of these ligases has been consistently observed in models of sarcopenia and immobilization-induced muscle atrophy, and pharmacological interventions that suppress UPS-related gene expression have shown efficacy in mitigating muscle loss (Shin et al., 2020; Jeon and Choung, 2021). In the present study, HI significantly reduced the phosphorylation of FOXO3a, accompanied by a marked increase in the protein expression of Atrogin-1 and MuRF1, indicating activation of the UPS. Notably, LGA2 treatment restored FOXO3a phosphorylation and suppressed the upregulation of Atrogin-1 and MuRF1, thereby inhibiting proteolytic activity associated with immobilization.

Myogenesis consists of two distinct stages: myoblast proliferation and differentiation, both orchestrated by myogenic regulatory factors such as MyoD, myogenin, and MyHC (Zammit, 2017). Among these, MyHC not only serves as a marker of terminal differentiation but also determines muscle fiber type (Augusto et al., 2004). Skeletal muscle fibers are classified into slow-twitch (type I) and fast-twitch (type II) subtypes based on MyHC isoform expression. Type I fibers express MyHC I and rely on oxidative metabolism, whereas type II fibers—including IIa, IIx, and IIb—utilize glycolytic pathways to varying extents (Talbot and Maves, 2016). Elevated Atrogin-1 expression under atrophic conditions has been shown to suppress myogenesis by targeting MyoD and Myogenin for polyubiquitination and degradation, thereby impairing the transcriptional regulation of muscle differentiation (Bodine and Baehr, 2014; Tintignac et al., 2005). In addition, MuRF1 facilitates proteasome-mediated degradation of MyHC through direct ubiquitination, contributing to the loss of structural muscle proteins and fiber shrinkage (Fanzani et al., 2012). In the present study, immobilization led to marked reductions in MyHC isoforms and Myogenin expression, confirming transcriptional suppression of the myogenic program. LGA2 supplementation significantly restored the expression of MyHC IIa, IIx, and IIb, and reversed the downregulation of Myogenin, suggesting a protective effect on myogenic potential under atrophic conditions.

Inflammatory signaling is increasingly recognized as a critical contributor to disuse-induced skeletal muscle atrophy (Sartori et al., 2021; Gao et al., 2018). Chronic low-grade inflammation elevates pro-inflammatory cytokines such as IL-6 and TNF-*α*, which activate catabolic pathways including NF-κB and FOXO3a, thereby promoting proteolysis via the upregulation of atrogenes such as Atrogin-1 and MuRF1 (Shen et al., 2024). These cytokines not only disrupt muscle protein homeostasis, but also impair mitochondrial function and contribute to oxidative stress, further exacerbating muscle loss (Costamagna et al., 2015; Bonetto et al., 2012). Moreover, persistent inflammation has been shown to interfere with the differentiation of satellite cells by suppressing key myogenic transcription factors, including MyoD and Myogenin, through STAT3 and NF-κB signaling pathways (Hoene et al., 2013; Pérez-Baos et al., 2018). In line with these findings, the present study showed that HI significantly increased local and systemic inflammatory markers, including TNF-α, IL-6, iNOS, and COX-2, while concurrently decreasing the expression of the anti-inflammatory cytokine IL-10. These inflammatory changes were accompanied by suppressed expression of Myogenin and MyHC isoforms, indicating that disuse-induced inflammation negatively impacted myogenic differentiation as well as protein homeostasis. However, oral administration of LGA2 markedly attenuated the inflammatory response in both muscle and serum, downregulating pro-inflammatory cytokines and restoring IL-10 expression. This anti-inflammatory effect was paralleled by enhanced expression of MyHC and Myogenin, suggesting that LGA2 may mitigate inflammation-mediated suppression of myogenesis. Together with its ability to inhibit key mediators of proteolysis, these findings underscore the dual anti-catabolic and pro-myogenic potential of LGA2 in HI-induced sarcopenia.

In addition to these local and systemic effects, LGA2 may also exert its actions through the gut–muscle axis. Probiotics, including various *Lactobacillus* species, have been reported to modulate gut microbiota composition, enhance intestinal barrier function, and influence nutrient utilization, thereby indirectly affecting muscle protein turnover and systemic inflammatory status (Zhu et al., 2025). Although gut microbiota profiles and intestinal parameters were not assessed in this study, such indirect pathways cannot be excluded and may represent an additional mechanism contributing to the protective effects of LGA2 in disuse-induced sarcopenia.

While this study demonstrates the protective role of *L. gasseri* LGA2 against disuse-induced muscle atrophy, several aspects warrant further investigation. Although *in vitro* screening confirmed LGA2’s functional efficacy, its direct molecular targets within muscle cells remain to be identified. This study revealed that LGA2 restores FOXO3a phosphorylation to suppress ubiquitin–proteasome-mediated protein degradation; however, additional analyses of upstream PI3K/Akt/mTOR components (e.g., Akt, mTOR phosphorylation) are needed to refine this mechanism. Future investigations should determine whether the systemic effects of LGA2 arise predominantly from muscle-associated signaling pathways or involve additional physiological systems not examined in the present study. Taken together, these findings support the potential of LGA2 as a probiotic candidate for mitigating disuse-induced muscle atrophy, in part through modulation of the FOXO3a signaling axis.

## 5 Conclusion

In this study, we first screened various *Lactobacillus* strains *in vitro* based on their ability to suppress muscle protein degradation and inflammation. Among the candidates, LGA2 was selected for its strong anti-catabolic and anti-inflammatory potential. Whole genome analysis further revealed the presence of genetic features associated with peptide transportation and antioxidative functions, supporting its functional relevance. Subsequent *in vivo* experiments using a HI-induced muscle atrophy model demonstrated that LGA2 effectively preserved muscle mass and strength, suppressed proteolytic and inflammatory responses, and restored key myogenic factors. Collectively, these findings suggest that LGA2 is a promising probiotic strain for alleviating disuse-induced sarcopenia and may hold translational potential as a functional probiotic for maintaining muscle health in humans.

## Data Availability

The datasets presented in this study can be found in online repositories. The names of the repository/s and accession number(s) can be found at: PRJNA948172 (SRA).
