# Supplementary material for: Lactobacillus gasseri CBT LGA2 alleviates muscle protein degradation and inflammation in immobilization-induced mouse
Source: Front Microbiol. 2026 Jan 7;16:1728172. doi: 10.3389/fmicb.2025.1728172 (PMC12819605; doi:10.3389/fmicb.2025.1728172)
Supplement: Supplementary file 1 [file Table_1.DOCX]

**Supplementary Figure 1.** Body weight changes during the 3-week experimental period in hindlimb-immobilized mice. Data represent mean ± SD of 8 biological replicates (individual mice) per group.

**
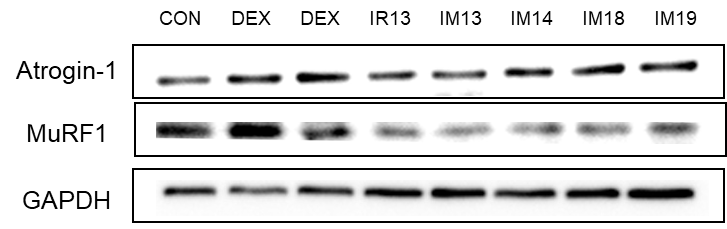
**

**Supplementary Figure 2.** Representative Western blot images from additional biological replicates for the analyses presented in Fig. 2A

**
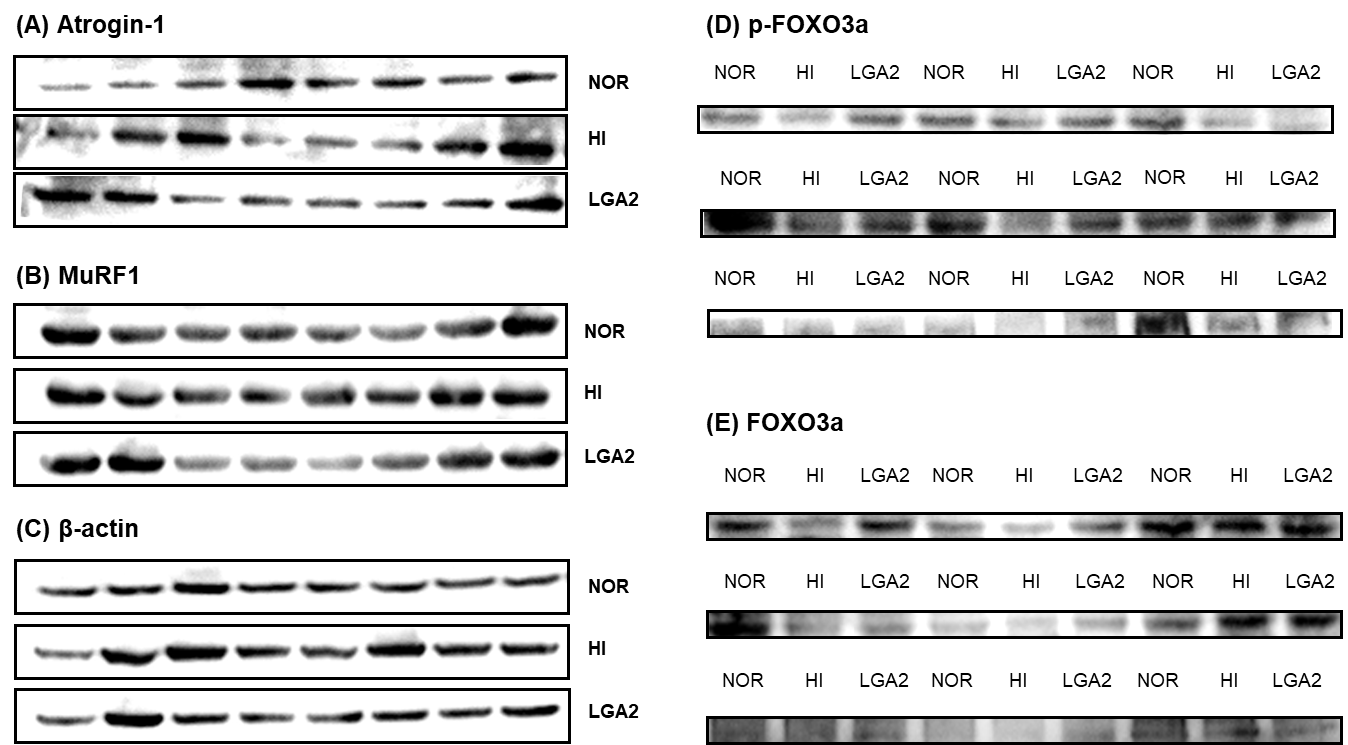
**

**Supplementary Figure 3.** Representative Western blot images from additional biological replicates for the analyses presented in Fig. 7A. (A) Atrogin-1 (B) MuRF1 (C) β-actin (D) p-FOXO3a and (E) FOXO3a.

**Supplementary table 1. Primer used in this study**

| **Gene name** | **Sequence (5’ – 3’)** | |
| --- | --- | --- |
| COX-2 | Forward | TTGAAGACCAGGAGTACCGC |
|  | Reverse | GGTACAGTCCCATGACATCG |
| iNOS | Forward | TTCCAGAATCCCTGGACAAG |
|  | Reverse | TGGTCAAACTCTTGGGGTTC |
| TNF-α | Forward | CTGAACTTCGGGGTCATCG |
|  | Reverse | GGCTTGTCACTCGAATTTTGAGA |
| IL-10 | Forward | CTTACTGACTGGCATGAGGATCA |
|  | Reverse | GCAGCTCTAGGAGCATGTGG |
| IL-1β | Forward | TGCCACCTTTTGACAGTGATG |
|  | Reverse | TTGGAAGCAGCCCTTCATCTT |
| IL-6 | Forward | AAGTCGGAGGCTTAATTACACATGT |
|  | Reverse | CCATTGCACAACTCTTTTCTCATTC |
| GAPDH | Forward | ACTCCACTCACGGCAAATTCA |
|  | Reverse | CGCTCCTGGAAGATGGTGAT |
